# The Cystine Knot Is Responsible for the Exceptional Stability of the Insecticidal Spider Toxin ω-Hexatoxin-Hv1a

**DOI:** 10.3390/toxins7104366

**Published:** 2015-10-26

**Authors:** Volker Herzig, Glenn F. King

**Affiliations:** Institute for Molecular Bioscience, The University of Queensland, St. Lucia QLD 4072, Australia; E-Mails: v.herzig@imb.uq.edu.au (V.H.); glenn.king@imb.uq.edu.au (G.F.K.); Tel.: +61-7-3346-2018 (V.H.); +61-7-3346-2025 (G.F.K.); Fax: +61-7-3346-2021 (V.H. & G.F.K)

**Keywords:** inhibitor cystine knot, physicochemical stability, spider toxin, insecticidal toxin, ω-hexatoxin-Hv1a, thermal stability, proteolytic degradation

## Abstract

The inhibitor cystine knot (ICK) is an unusual three-disulfide architecture in which one of the disulfide bonds bisects a loop formed by the two other disulfide bridges and the intervening sections of the protein backbone. Peptides containing an ICK motif are frequently considered to have high levels of thermal, chemical and enzymatic stability due to cross-bracing provided by the disulfide bonds. Experimental studies supporting this contention are rare, in particular for spider-venom toxins, which represent the largest diversity of ICK peptides. We used ω-hexatoxin-Hv1a (Hv1a), an insecticidal toxin from the deadly Australian funnel-web spider, as a model system to examine the contribution of the cystine knot to the stability of ICK peptides. We show that Hv1a is highly stable when subjected to temperatures up to 75 °C, pH values as low as 1, and various organic solvents. Moreover, Hv1a was highly resistant to digestion by proteinase K and when incubated in insect hemolymph and human plasma. We demonstrate that the ICK motif is essential for the remarkable stability of Hv1a, with the peptide’s stability being dramatically reduced when the disulfide bonds are eliminated. Thus, this study demonstrates that the ICK motif significantly enhances the chemical and thermal stability of spider-venom peptides and provides them with a high level of protease resistance. This study also provides guidance to the conditions under which Hv1a could be stored and deployed as a bioinsecticide.

## 1. Introduction

**Figure 1:**
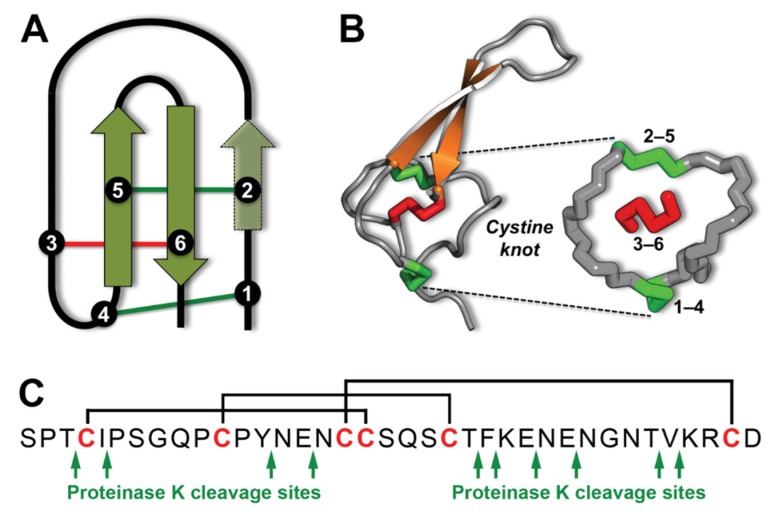
Inhibitor cystine knot of the spider-venom peptide ω-hexatoxin-Hv1a. (**A**) Schematic of the ICK motif, which comprises an antiparallel β sheet stabilised by a cystine knot [1]. β strands are shown in green and the six cysteine residues that form the cystine knot are labeled 1–6. In spider toxins, the β sheet typically comprises only the two β strands housing Cys5 and Cys6, although a third *N*-terminal strand encompassing Cys2 is sometimes present [3]. The two “outer” disulfide bonds are shown in green and the “inner” disulfide bridge is red. (**B**) Schematic of the three-dimensional structure of the 37-residue spider-venom peptide Hv1a (PDB 1AXH) [5] highlighting the ICK motif. The cystine knot comprises a ring formed by two disulfides (Cys1–Cys4 and Cys2–Cys5, green) and the intervening sections of polypeptide backbone (gray), with a third disulfide (Cys3–Cys6, red) piercing the ring to create a pseudo-knot. The hydrophobic core of the toxin consists primarily of the two central disulfide bridges connected to the β strands. (**C**) Primary structure of Hv1a showing the location of the three disulfide bonds and the 10 proteinase K cleavage sites predicted by PeptideCutter [6].

The inhibitor cystine knot (ICK) is a protein scaffold defined as an antiparallel β sheet stabilized by a cystine knot [1,2]. The β sheet typically comprises two β strands, although a third *N*-terminal strand is sometimes present [3] (Figure 1A). The cystine knot itself comprises a ring formed by two disulfide bridges (Cys1–Cys4 and Cys2–Cys5) and the intervening sections of peptide backbone, with a third disulfide bond (Cys3–Cys6) penetrating the ring to create a pseudo-knot (Figure 1B). The two central disulfide bridges emanating from the β strands are tightly packed against one another and they form the compact hydrophobic core of ICK peptides [4].

Peptides containing an ICK motif are found in taxonomically diverse organisms ranging from fungi and plants to molluscs and arthropods [1,7], although they are most abundant in the venom of spiders [8]. ICK-containing peptides are frequently considered to be highly stable over a wide range of physicochemical conditions, but very few studies have experimentally examined their stability. The best-studied example is kalata B1, a plant cyclotide whose thermal, chemical and enzymatic stability has been examined in detail. Remarkably, it was demonstrated that the cystine knot is more important for the stability of this cyclic peptide than its circular backbone [9].

The ICK motif is the most common protein architecture found in spider-venom peptides [8], but data on the contribution of this motif to the stability of spider toxins is lacking. Thus, in the present study, we used the insecticidal spider-venom peptide ω-hexatoxin-Hv1a (Hv1a) as a model ICK peptide and explored the contribution of the cystine knot to its physicochemical stability. Hv1a is 37-residue ICK peptide isolated from the venom of the lethal Australian funnel-web spider *Hadronyche versuta* (Figure 1C). It has been used as a lead for bioinsecticide development due to its ability to selectively block invertebrate, but not vertebrate, voltage-gated calcium (Ca_V_) channels [5,8,10,11,12] and its lack of toxicity to bees [13]. Hv1a has high level of biological stability as it is active when expressed *in planta* [8,14] and it is capable of crossing the insect blood brain barrier in order to inhibit Ca_V_ channels in the insect central nervous system [5,15]. The molecular basis of Hv1a’s inherent biological stability is presumed to be its ICK motif but this has not been experimentally demonstrated.

In order to determine how the cystine knot contributes to the physicochemical stability of Hv1a, we compared native Hv1a with reduced and alkylated (linear) Hv1a over a wide range of temperatures, solvents and pH conditions, and after incubation in proteinase K, insect hemolymph, and human plasma. We show that Hv1a has a high level of chemical and thermal stability and that it is highly resistant to proteolytic degradation. Reduction and alkylation of the six cysteine residues that form the cystine knot motif of Hv1a completely abrogated the peptide’s resistance to enzymatic degradation and its resistance to high temperature and acidic pH. Even the long-term stability of Hv1a in water and organic solvents was compromised by loss of the ICK motif. This study reveals that the ICK motif is capable of providing spider-venom peptides with a high level of thermal, chemical and biological stability, and it further highlights the ICK motif as an excellent scaffold for protein engineering studies directed towards the development of peptide drugs and bioinsecticides.

## 2. Results and Discussion

### 2.1. Thermal Stability

**Figure 2:**
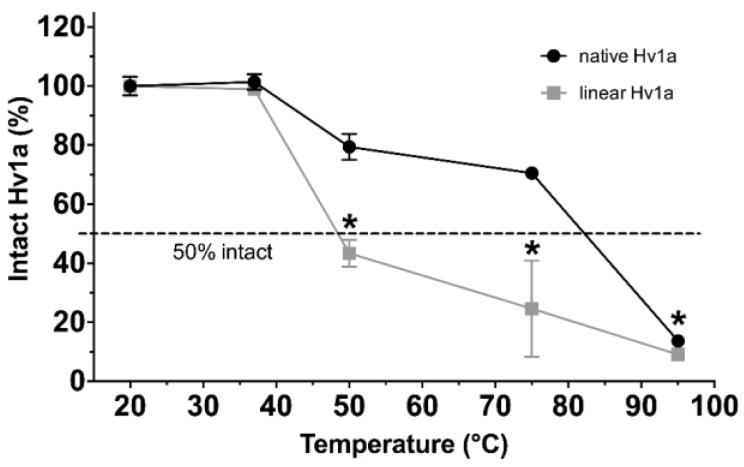
Thermal stability of Hv1a. Fraction of intact native Hv1a (black circles) and linear Hv1a (grey squares), relative to the corresponding 20 °C samples, after incubation at the indicated temperatures for 24 h. The dashed line indicates that the *T*_m_ under these conditions is ~48 °C and ~83 °C for linear and native Hv1a, respectively. Data are mean ± standard deviation (SD). In this and subsequent figures, asterisks indicate statistically significant differences between native and linear Hv1a (*p* < 0.05).

Figure 2 summarises the stability of Hv1a when incubated for 24 h over a temperature range from 20 to 95 °C. There was no visible effect on toxin stability for both native and linear Hv1a up to 37 °C (Figure 2), but peptide stability decreased at a much faster rate for linear Hv1a at temperatures exceeding 37 °C. Remarkably, ~71% of native Hv1a remained intact after incubation for 24 h at 75 °C, whereas only ~25% of linear Hv1a remained intact under these conditions. In order to determine whether the degradation observed at high temperature is reversible, we incubated a sample of Hv1a at 95 °C for 24 h, then stored the sample at 20 °C for 3 days prior to HPLC analysis. There was no recovery in the level of intact peptide over the 3 days (data not shown), indicating that the degradation observed at 95 °C is irreversible. Nevertheless, based on the data shown in Figure 2, Hv1a should be highly stable in the field even if employed as a bioinsecticide in climates that reach high temperatures.

### 2.2. pH Stability

**Figure 3:**
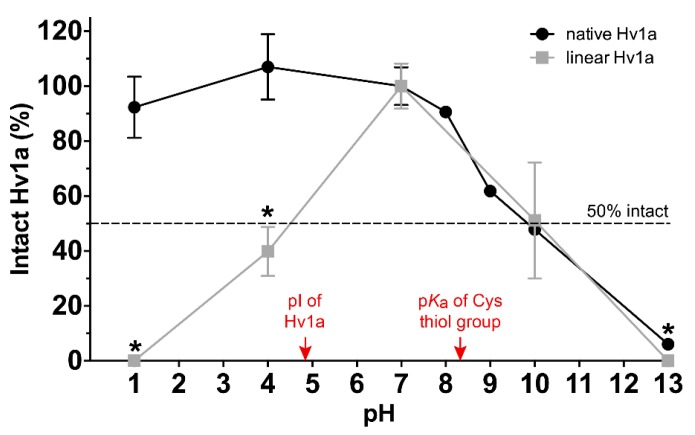
pH stability of Hv1a. Fraction of intact native Hv1a (black circles) and linear Hv1a (grey squares), relative to the corresponding pH 7 samples, following incubation for 24 h at the indicated pH. Dashed line corresponds to 50% intact toxin. The pI of Hv1a and the *pK*a of free cysteine thiol groups are indicated by the arrows. Data are mean ± SD.

Figure 3 shows the stability of Hv1a when incubated for 24 h in buffers with pH ranging from 1 to 13. Native Hv1a was highly stable at neutral and acidic pH, with essentially no degradation between pH 1 and 7. However, the peptide began to degrade as the pH approached the p*K*_a_ of cysteine (~8.3), and degradation became very apparent at pH 9 and above. Only 6% of the peptide remained intact after 24 h at pH 13 (Figure 3). Additional peaks with higher retention time than Hv1a began to emerge in the RP-HPLC chromatograms of samples incubated at pH 8 and higher (data not shown). MALDI mass spectrometric analysis revealed that these peaks have the same mass as Hv1a and we therefore conclude that these peaks correspond to isoforms of Hv1a resulting from disulfide-bond shuffling at alkaline pH. In contrast with native toxin, the linear version of Hv1a was only stable at neutral pH values, with marked degradation under both acidic and alkaline conditions. This instability presumably arises from well-known processes that impact on protein stability such as asparagine deamidation, aspartate isomerisation, and racemisation [16,17]. Interestingly, Hv1a contains an Asn-Gly sequence (Figure 1), which has been shown to be highly susceptible to asparagine deamidation [18].

The pH of the gut lumen in insects is highly variable, although there is a general trend, particularly in exopterygous insects, towards acidic crops, neutral to mildly alkaline midguts, and neutral to acidic hindguts [19,20]. Hv1a is stable under all of these pH conditions. However, several orders of endopterygote insects such as lepidopterans, coleopterans, and dipterans have highly alkaline midguts (pH > 8) [19,20,21]. This is especially true of lepidopteran larvae that feed on the leaves of trees, where the average midgut pH of ~8.7 is believed to provide protection against leaf tannins and two-component plant chemical defenses [21,22]. The instability of Hv1a at pH > 8 may therefore compromise its effectiveness against lepidopterans and other insects with highly alkaline guts. Nevertheless, it has been shown that transgenic expression of Hv1a in tobacco and cotton provides high levels of resistance against larvae of the lepidopterans *Helicoverpa armigera* (cotton bollworms) and *Spodoptera littoralis* (cotton leafworms) [23,24,25]. Thus, Hv1a must be sufficiently stable within the gut of these lepidopterans that enough toxin is taken up into the hemolymph to cause toxic effects at target sites (*i.e.*, Ca_V_ channels) within the central nervous system.

### 2.3. Chemical Stability

**Figure 4:**
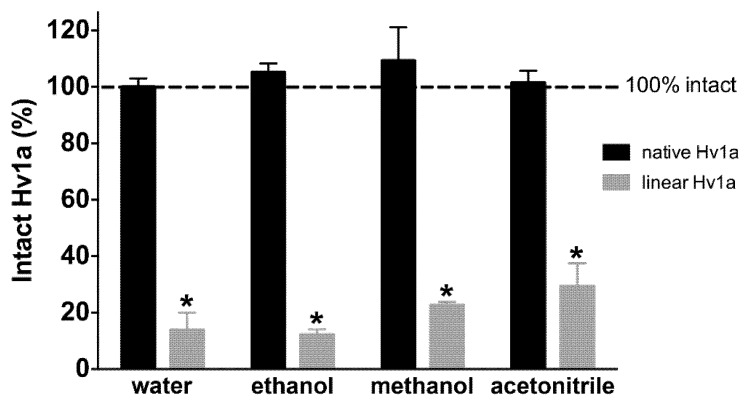
Stability of Hv1a in various solvents. Fraction of intact native Hv1a (black columns) and linear Hv1a (grey columns) after incubation for seven days in water and organic solvents. Toxin amounts were quantified relative to a control sample incubated for one day in water. Dashed line indicates 100% intact toxin. Data are mean ± SD.

Figure 4 shows that native Hv1a is highly stable during long-term incubation at room temperature (in the dark) in water and different organic solvents (*i.e.*, ethanol, methanol and acetonitrile). In contrast, linear Hv1a was unstable over one week in water and all three organic solvents, although stability was marginally higher in acetonitrile. This further highlights the importance of the cystine knot for the long-term stability of ICK peptides. The long-term stability of Hv1a in organic solvents is useful from a bioinsecticide development perspective since insecticides are often formulated with emulsifiers, stabilisers, surfactants or other adjuvants that require dissolution in organic solvents [26].

### 2.4. Proteolytic Stability

**Figure 5:**
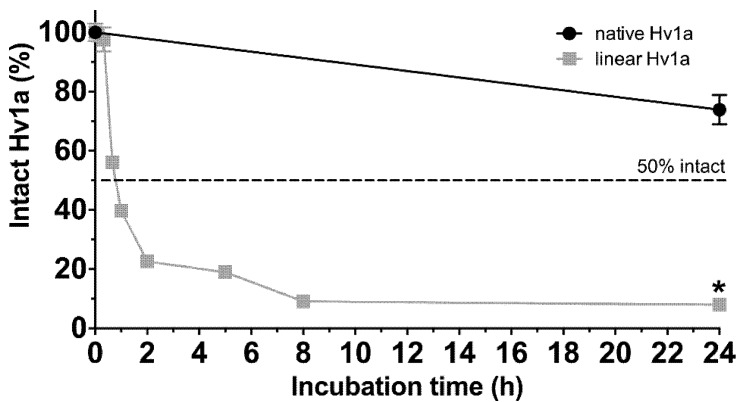
Proteolytic stability of Hv1a. Fraction of intact native Hv1a (black circles) and linear Hv1a (grey squares) after incubation with proteinase K (1:200 molar ratio) at pH 7.5 and 37 °C for up to 24 h. The values for native Hv1a were quantified relative to a sample of native Hv1a incubated for 24 h under the same conditions without proteinase K. Values for linear Hv1a were quantified relative to a sample of linear Hv1a at pH 7.5 incubated for 0 h without proteinase K. The dashed line indicates 50% intact toxin. Data are mean ± SD.

Proteinase K is a broad-spectrum serine protease from the fungus *Engyodontium album* that primarily cleaves at peptide bonds on the *C*-terminal side of aliphatic and aromatic amino acid residues. It is commonly used to remove contaminating protein from nucleic acid preparations [27], and consequently it provides a stringent test of peptide/protein stability. Hv1a is predicted to contain 10 proteinase K cleavage sites dispersed along the entire length of the toxin (see Figure 1C). Thus, as expected, linear Hv1a was highly susceptible to proteinase K cleavage, with less than 25% of the peptide remaining intact after incubation for just 2 h at 37 °C using a 1:200 molar ratio of proteinase K:Hv1a (Figure 5, grey squares). In striking contrast, 74% of native Hv1a remained intact after incubation for 24 h under the same conditions (Figure 5, black circles). Thus, the ICK motif of Hv1a provides a very high level of resistance against the proteolytic activity of proteinase K.

### 2.5. Stability in Insect Hemolymph

**Figure 6:**
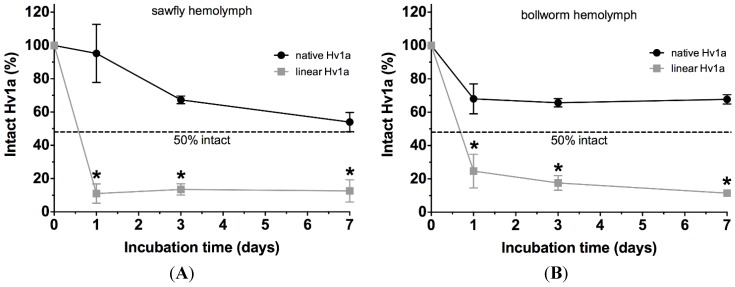
Stability of Hv1a in insect hemolymph. Fraction of intact native Hv1a (black circles) and linear Hv1a (grey squares) after incubation in (A) sawfly hemolymph and (B) bollworm hemolymph for up to seven days at ambient temperature (~20 °C). Native and linear Hv1a were quantified relative to control samples that were incubated in water for one day. The dashed line indicates 50% intact toxin. Data are mean ± SD.

Since Hv1a is considered a lead peptide for bioinsecticide development [8,13], its stability in insect gut and hemolymph is critical in addition to its environmental stability. We therefore examined the stability of Hv1a in hemolymph extracted from sawfly larvae and cotton bollworms (*i.e.*, larvae of the recalcitrant lepidopteran pest *Helicoverpa armigera*). Most insects carefully regulate the pH of their hemolymph, typically in the range 6.4–6.8 [19,28,29]. The pH of the extracted sawfly hemolymph was measured to be 6.68, which is similar to the pH of 6.99 reported for hemolymph from larvae of the European pine sawfly *Neodiprion sertifer* [30]. The hemolymph extracted from *H. armigera* larvae had a pH of 6.45, which is slightly lower than the pH of 6.8 reported for hemolymph from larvae of the related lepidopteran *H. zea* [31]*.* Although native Hv1a is highly stable at neutral and acidic pH (see Figure 3), insect hemolymph contains proteases that can degrade exogenous peptides [32]. Nevertheless, we found that Hv1a is highly stable in insect hemolymph, with 54%–68% of the peptide remaining intact after seven days incubation at ambient temperature (~20 °C) in hemolymph from sawfly larvae (Figure 6A) and bollworms (Figure 6B). In contrast, only 11%–25% of linear Hv1a remained intact after one day of incubation with sawfly or bollworm hemolymph (Figure 6A,B, grey squares), again highlighting the importance of the cystine knot for providing resistance against chemical degradation and proteases.

Unfortunately, we were unable examine toxin stability in insect midgut solution, as we found it difficult to isolate midgut contents without contamination from foregut, hindgut or hemolymph.

**Figure 7:**
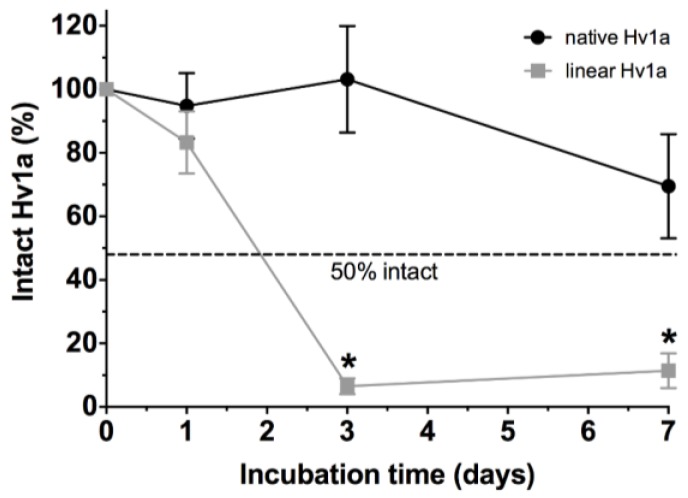
Stability of Hv1a in human plasma. Fraction of intact native Hv1a (black circles) and linear Hv1a (grey squares) after incubation in human plasma for up to seven days at ambient temperature (~20 °C). Native and linear Hv1a were quantified relative to control samples that were incubated in water for one day. The dashed line indicates 50% intact toxin. Data are mean ± SD.

### 2.6. Stability in Human Plasma

Remarkably, we found that Hv1a is even more stable in human plasma than insect hemolymph, with ~70% of the peptide remaining intact after seven days incubation in plasma at ~20 °C (Figure 7, black circles). In contrast, only ~7% of linear Hv1a remained intact after three days incubation in human plasma (Figure 7, grey squares). Thus, the cystine knot of Hv1a provides exceptional resistance against plasma proteases as well as plasma reductants such as glutathione and serum albumin that can potentially reduce disulfides add/or cause disulfide scrambling [33].

### 2.7. Comparison of Hv1a Stability with Other ICK Peptides

The ICK motif is an unusual protein architecture in which a single disulfide bond is threaded through a closed loop formed by two other disulfide bonds and the intervening sections of the polypeptide backbone [1,3,7,34]. ICK peptides, also known as knottins [34], have evolved independently in a diverse range of terrestrial taxa, including insects, arachnids, fungi, and plants [7], as well as marine cone snails [1], sponges [35], horseshoe crabs [36] and sea anemones [37]. This protein fold appears to have been evolutionarily favoured for two reasons. First, the ICK motif is highly plastic to sequence changes [38,39], which allows it to support a wide variety of disparate functions [7]. Second, although it is not a true knot in the mathematical sense, the inhibitor cystine knot is expected to greatly reduce the propensity for peptide unfolding, and hence it is generally thought to imbue ICK peptides with a high level of physicochemical stability [4,7,40].

Surprisingly, very few studies have examined the stability of native knottin peptides in detail, much less addressed whether the cystine-knot motif is the underlying cause of unusually high levels of chemical, thermal and biological stability. The only systematic study of this kind was performed on kalata B1, a plant cyclotide [9]. Cyclotides are macrocyclic plant knottins containing a head-to-tail cyclised peptide backbone in addition to a cystine knot motif. Kalata B1 was shown to be highly thermostable and extremely resistant to chaotropic agents and proteases. These properties were largely retained in an acyclic permutant but not when the disulfide bonds were eliminated by reduction and alkylation, which led the authors to conclude that the cystine knot, rather than backbone cyclization, is the primary contributor to the remarkable stability of kalata B1 [9]. The same authors demonstrated that native conotoxin PVIIA, a knottin peptide from the venom of a marine cone snail, is impervious to various endoproteinases but this protease resistance is lost when the cystine knot is eliminated by disulfide reduction [9]. Engineered ICK peptides derived from plant knottins or the cystine-knot motif of human agouti-related protein were shown to be highly resistant to pepsin and elastase, and have long half-lives in rat plasma, but the contribution of the cystine knot to these properties was not examined [41,42]. A variety of diverse ICK peptides from sponges [35] and spiders [32] have been shown to be resistant to proteases and/or have high levels of stability in human plasma or insect hemolymph, but the contribution of the cystine-knot motif to this stability was not examined.

The current work represents the first systematic study of the contribution of the inhibitor cystine knot to the thermal, chemical, and biological stability of a native, non-cyclic knottin. We demonstrated that the cystine knot underlies the remarkable resistance of the insecticidal toxin Hv1a to extremes of temperature, acidic pH, organic solvents, and both human plasma and insect hemolymph. Highly alkaline pH (>8) was the only condition under which native Hv1a was not considerably more stable than its linearised counterpart, presumably due to reduction/scrambling of the disulfide bonds in the cystine knot as the pH exceeds the p*K*_a_ of the cysteine side-chain thiol groups. OAIP-1, an insecticidal knottin from tarantula venom, was also found to be susceptible to degradation under alkaline conditions [32].

Hv1a was isolated from the venom of the Australian funnel-web spider *Hadronyche versuta*, a close relative of the infamous Sydney funnel-web spider *Atrax robustus* that is lethal to rodents and primates [43]. The lethal component of *A. robustus* venom is the knottin peptide δ-hexatoxin-Ar1a [44,45,46], which potently delays inactivation of voltage-gated sodium channels [47]. Thus, data from 1961 on the stability of crude *A. robustus* venom [43] can be considered a proxy for the stability of δ-hexatoxin-Ar1a. Wiener found that the toxicity to mice of *A. robustus* venom was not altered after storage for four months at 4 °C, incubation at 37 °C for two days, incubation at 100 °C for 1 h, incubation at 100 °C for 20 min in the presence of 0.1 M HCl (pH~1), storage for 14 days in 33% acetic acid, and incubation with 0.1% pepsin and 0.2% HCl at 40 °C for 2 h [43]. However, incubation of venom for 24 h at pH 8.5 and 37 °C resulted in a slight loss of activity, incubation for 2 h at 40 °C with 0.1% trypsin and 0.1% Na_2_CO_3_ (pH ~11) resulted in a 50% loss of toxicity, and activity was completely lost when venom was incubated with 0.1 M NaOH (pH~13) for 20 min at 100 °C [43]. Finally, Wiener found that although *A. robustus* venom precipitates in 80% ethanol, the precipitate readily dissolves in water and retains toxicity [43]. Thus, *A. robustus* venom, and by extension the lethal knottin peptide δ-hexatoxin-Ar1a, is stable under the same conditions as Hv1a: it is resistant to extremes of temperature, acidic pH, ethanol, and proteases, but is susceptible to degradation under highly alkaline conditions. We conclude that these are likely to be general properties of spider-venom knottins.

## 3. Experimental Section

### 3.1. Chemicals

2-Aminoethanol and triethylphosphine were from Sigma-Aldrich (Castle Hill, NSW, Australia). 2-Iodoethanol was from Acros Organics (Thermo Fisher Scientific, Geel, Belgium). Proteinase K was from Promega (Madison, WI, USA) and synthetic Hv1a was kindly supplied by Vestaron Corporation (Kalamazoo, MI, USA).

### 3.2. Sample Treatment

Every condition for each treatment was tested in triplicate for both native and linear Hv1a, and all data reported are mean ± SD. Unless otherwise stated, all samples were immediately frozen after treatment and then analysed via HPLC. Native Hv1a was linearised by one-step reduction and alkylation (RA) of the six Cys residues [48]. Toxin (1 mg) was dissolved in 450 µL of water prior to addition of 5 µL aminoethanol, 10 µL iodoethanol, 2 µL triethylphosphine (0.1 M solution in tetrahydrofuran), and 450 µL acetonitrile. After incubation at 37 °C for 2 h, the toxin sample was dried by vacuum centrifugation and the fully reduced and alkylated toxin was purified using RP-HPLC on a Shimadzu 20A series HPLC system (Shimadzu Scientific Instruments, Rydalmere, NSW, Australia).

All native Hv1a samples were processed using a reverse-phase analytical column (Jupiter C_18_, 5 µm particle size, 300 Å pore size, 150 × 4.6 mm; Phenomenex, Lane Cove, NSW, Australia;) on a Shimadzu 20A series HPLC system, with detection at 214 nm. Solvent A (0.1% formic acid in water) and solvent B (0.1% formic acid in 90% acetonitrile) were used at a flow rate of 1 mL/min using a gradient of 5% solvent B for the first 3 min, 5%–25% solvent B over the next 10 min, then 25%–80% solvent B over 0.5 min. For all linear Hv1a samples, a VisionHT HILIC column (5 µm particle size, 150 × 4.6 mm; Grace, Columbia, MD, USA) was used on a Shimadzu 20A series HPLC system with HILIC-solvent A (0.05% triflouracetic acid (TFA) in water) and HILIC-solvent B (90% acetonitrile, 0.043% TFA in water). The following gradients were used for the HILIC runs: 85% HILIC solvent B for the first 3 min, then 85%–55% HILIC solvent B for another 6 min, then 55%–5% HILIC solvent B for 1 min followed by 5% HILIC solvent B for 1 min. Peptide peaks (identified from absorbance at 214 nm) were collected manually and molecular masses determined using MALDI mass spectrometry on a 4700 Proteomics Analyzer (Applied Biosystems, Foster City, CA, USA) using α-cyano-4-hydroxycinnamic acid (CHCA) as matrix. The fractions containing native or linear Hv1a were identified based on molecular mass and retention time, then the amount of intact toxin after each treatment was quantified based on peak area.

### 3.3. Thermal Stability

Thermal stability was examined by incubating native (24.7 µM) and linear Hv1a (18.7 µM) for 24 h at various temperatures (20, 37, 50, 75 and 95 °C). Following the respective incubation period, samples were immediately stored at −20 °C prior to HPLC fractionation. In order to determine whether the effect of high temperature is reversible for the native Hv1a, a sample was incubated for 24 h at 95 °C and then stored at room temperature for three days before HPLC analysis. All native and linear Hv1a amounts were quantified relative to their respective 20 °C sample.

### 3.4. pH Stability

Stability under varying pH conditions was assessed by incubating native (49.4 µM) and linear Hv1a (18.7 µM) for 24 h at room temperature in buffers of the following pH/composition (p*K*_a_ values for the relevant species are indicated): (i) pH 1 (50 parts 0.2 M KCl plus 134 parts 0.2 M HCl); (ii) pH 4 (0.2 M sodium acetate, p*K*_a_ 4.76); (iii) pH 7 (100 parts 0.1 M KH_2_PO_4_ plus 58.2 parts 0.1 M NaOH; p*K*_a2_ 7.21); (iv) pH 8 (100 parts 0.1 KH_2_PO_4_ plus 93.4 parts 0.1 M NaOH; pK_a2_ 7.21); (v) pH 9 (0.1 M Tris-HCl, p*K*_a_ 8.07); (vi) pH 10 (966.4 parts 0.1 M Na_2_HPO_4_ plus 33.6 parts 0.1 M NaOH); (vii) pH 13 (50 parts 0.2 M KCl plus 132 parts 0.2 M NaOH). After the incubation period, native Hv1a samples were desalted using a Maxi-Clean SPE column (Large Pore C18; Grace, Columbia, MD, USA) before application onto the C_18_ HPLC column. For desalting, 10 mL of 5% solvent B was used to remove salts then Hv1a was eluted with 10 mL of 45% solvent B and lyophilised prior to RP-HPLC analysis. All native and linear Hv1a amounts were quantified relative to their respective pH 7 sample.

### 3.5. Chemical Stability

The long-term stability of native (49.4 µM) and linear Hv1a (18.5 µM) in water and organic solvents was determined by incubation in Milli-Q water, methanol, ethanol or acetonitrile in the dark for seven days at room temperature. All samples were then lyophilised before HPLC analysis. The amounts of native and linear Hv1a were quantified relative to a respective control sample that was incubated in Milli-Q water for one day.

### 3.6. Proteolytic Stability

Analysis of the Hv1a sequence using PeptideCutter [6] indicated that it contains more potential cleavage sites for proteinase K than any other protease examined (Figure 1C). Accordingly, native Hv1a (24.7 µM) was incubated in a pH 7.5 buffer (0.2 M phosphate, 5 mM CaCl_2_) with proteinase K added at a molar ratio of 1:200 (proteinase K:Hv1a). Samples were then incubated at 37 °C for 24 h. An identical sample of native Hv1a without proteinase K was used as a negative control and also incubated at 37 °C for 24 h. This sample was used as a reference (*i.e.*, set to 100%) for the relative quantification of native Hv1a sample incubated in the presence of proteinase K. After the incubation period, native Hv1a samples were processed through a Maxi-Clean C_18_ column prior to HPLC (conditions see above under pH stability). Linear Hv1a (6.9 µM) was incubated in a pH 7.5 buffer (0.2 M phosphate, 5 mM CaCl_2_) with proteinase K added at a molar ratio of 1:200 (proteinase K:Hv1a) for a range of different time intervals (20 and 40 min, and 1, 2, 5, 8 and 24 h). All linear Hv1a amounts were quantified relative to a sample of linear Hv1a in the pH 7.5 buffer (without proteinase K) that was immediately processed through a Maxi-Clean C_18_ column prior to HPLC. All other samples were incubated for the respective incubation time before being cleaned through a Maxi-Clean C_18_ column.

### 3.7. Stability in Insect Hemolymph

Sawfly larvae (Hymenoptera, Pergidae) were collected from Regency Downs, Queensland, Australia. Fifth instar *H. armigera* larvae were purchased from AgBiTech Pty Ltd. (Clifford Gardens, Queensland, Australia). A 1.0 mL Terumo Insulin syringe (B-D Ultra-Fine, Terumo Medical Corporation, Somerset, NJ, USA) with a fixed 29 gauge needle was used to puncture the insect exoskeleton and collect the extruded hemolymph, which was then centrifuged at 4 °C for 10 min at 10,000 rpm to remove insoluble material. The pH of supernatant was measured using an Ultra M Micro Combination pH electrode (Van London Co., Houston, TX, USA). Native Hv1a (367 µM) and linear Hv1a (154 µM) were dissolved in clarified hemolymph, then the samples were incubated for 1–7 days at room temperature (~20 °C). Prior to HPLC analysis, all samples were processed through a Maxi-Clean SPE column. A HPLC chromatogram of a hemolymph-only control sample was subtracted from each HPLC chromatogram of the respective native or linear Hv1a samples in order to account for peaks originating from endogenous hemolymph compounds. All native and linear Hv1a amounts were quantified relative to a respective control sample that was incubated in Milli-Q water for one day.

### 3.8. Stability in Human Plasma

Native Hv1a (367 µM) or linear Hv1a (116 µM) were incubated for 1–7 days at room temperature (~20 °C) in pooled human plasma (Innovative Research, Novi, MI, USA). Native and linear Hv1a samples incubated in Milli-Q water for seven days at room temperature (~20 °C) were used as controls. Prior to HPLC analysis, samples were processed through a Maxi-Clean SPE column. Native and linear Hv1a were quantified relative to a respective control sample that was incubated in Milli-Q water for one day.

### 3.9. Statistical Analyses

GraphPad Prism 6 (GraphPad Software, La Jolla, CA, USA) was used to determine statistically significant differences between native and linear Hv1a for each treatment condition using multiple *t* tests followed by a Holm-Sidak correction. *p* < 0.05 was defined as statistically significant.

## 4. Conclusions

We demonstrated that the insecticidal spider-venom peptide Hv1a is remarkably resistant to chemical degradation; the peptide is stable over the pH range 1–8, at temperatures up to 75 °C, and when dissolved in a range of organic solvents. Moreover, we found that Hv1a is highly resistant to proteolytic degradation, with outstanding stability in insect hemolymph and human plasma, and when incubated with proteinase K. These high levels of chemical and proteolytic resistance were completely abolished when the toxin’s ICK motif was destroyed by reduction and alkylation of the six cysteine residues. We conclude that the ICK motif, which is prevalent in spider-venom peptides, provides these toxins with remarkable levels of chemical, thermal, and biological stability.

The current study also provides a guide to the range of conditions under which Hv1a could be formulated and used as a bioinsecticide. Our data show that formulations requiring dissolution in organic solvents and/or acidic conditions should not adversely affect the stability of Hv1a. Moreover, we demonstrated that Hv1a has a high level of thermal stability, which should facilitate its application in the field in high-temperature climates.
